# ATAD3A structurally links mtDNA replication and mitochondrial fission

**DOI:** 10.64898/2026.06.23.733767

**Published:** 2026-06-25

**Authors:** Nitish Dua, Boyuan Ma, Samantha Oviedo, Hamidreza Rahmani, Tumara Boyd, Donghyun Park, R. Luke Wiseman, Danielle A. Grotjahn

## Abstract

Mitochondrial function depends on the maintenance of its genome, and disruptions in copy number and distribution are hallmarks of mitochondrial disorders. Mitochondrial DNA (mtDNA) replication is spatially and temporally linked to mitochondrial division (i.e., fission). However, the signal that coordinates these two events, which are physically separated by the barrier of two mitochondrial membranes, remains unknown. To gain insight into this coordination, we employed correlative cryo-electron tomography (cryo-ET) to analyze the microenvironment surrounding replicating nucleoids. Mitochondrial regions containing replicating mtDNA exhibit a unique membrane architecture defined by the presence of clustered, membrane-spanning tethers that traverse the inner membrane space. Using a combination of superresolution microscopy and genetically encoded cryo-ET tagging technology, we identify these tethers as the AAA+ ATPase ATAD3A. We further show that ATAD3A knockdown reduces recruitment of the mitochondrial fission machinery, whereas overexpression promotes its recruitment and subsequent fission. Our work suggests that ATAD3A forms nanoscale linkages that coordinate these two distinct processes, revealing a new structural paradigm for organellar communication across distinct membrane-defined environments.

**Highlights:**

- Replicating mitochondrial DNA (mtDNA) nucleoids are surrounded by a distinct membrane microenvironment.
- ATAD3A forms membrane-spanning tethers enriched at replicating mtDNA sites.
- ATAD3A enrichment is necessary and sufficient to recruit mitochondrial fission machinery and induce fission at mtDNA replication sites.
- ATAD3A structurally couples mtDNA replication state to mitochondrial fission across distinct organellar subcompartments.

## Full Text Availability

The license terms selected by the author(s) for this preprint version do not permit archiving in PMC. The full text is available from the preprint server.

